# Usefulness of Cardiac Resynchronization Therapy Optimization Using Combined Electrocardiography and Echocardiography

**DOI:** 10.7759/cureus.78912

**Published:** 2025-02-12

**Authors:** Motomi Tachibana, Akihiro Hayashida, Yutaka Take, Kimikazu Banba, Akihisa Kimura, Tatsuya Shigematsu, Atsushi Hirohata

**Affiliations:** 1 Cardiology, Sakakibara Heart Institute of Okayama, Okayama, JPN

**Keywords:** cardiac resynchronization therapy, crt optimization, dyssynchrony, heart failure, speckle tracking echocardiography

## Abstract

Introduction

Optimization of cardiac resynchronization therapy (CRT) using a 12-lead electrocardiogram (ECG) alone may not sufficiently improve left ventricular (LV) function in all cases. Therefore, we aimed to investigate whether additional optimization using transthoracic echocardiography (TTE) could further enhance CRT efficacy.

Methods

Sixty-five patients who underwent CRT implantation between March 2018 and July 2022 at Sakakibara Heart Institute, Japan, were included in this study. Data were collected at three points: before optimization (point A), after ECG-based optimization (point B), and after TTE-based adjustments (point C).

Results

The mean age was 74±11 years (male: 78.4%). The PR interval was significantly prolonged at points B and C compared with that at point A. The QRS width narrowed significantly at point B (119±20 ms vs. 137±20 ms, p<0.01) but increased at point C (124±20 ms), approaching point A levels. Thirty-six patients achieved LV dyssynchrony improvement with ECG optimization alone, whereas 29 required TTE adjustments for further improvement. Patients needing TTE optimization had higher QRS width at point C than at point B. TTE adjustments significantly improved LV ejection fraction (28.1±6.8% to 31.5±8.0%, p=0.01) and reduced septal flash (46.2% to 15.4%, p=0.04). The need for TTE adjustments was similar to the usage of CRT devices with and without auto-adjustment functions.

Conclusion

TTE-based optimization enhances LV function and synchrony in cases where ECG-based adjustments alone are insufficient, highlighting the importance of TTE evaluation in CRT optimization.

## Introduction

Cardiac resynchronization therapy (CRT) improves outcomes in patients with heart failure (HF). However, approximately one-third of the patients do not fully benefit from CRT [1,2]. Notably, inappropriate atrioventricular interval (AVI)/ventriculo-ventricular interval (VVI) setting of pacing patients is responsible for the reduced efficacy of CRT. Post-implantation device optimization for better left ventricular (LV) function includes individualized programming of the AVI and VVI to maximize hemodynamic and clinical responses to CRT [3,4].

A 12-lead electrocardiogram (ECG) or transthoracic echocardiography (TTE) is the standard method for CRT optimization. However, recent reports have shown that ECG optimization is superior to TTE optimization. One advantage of ECG optimization is that it is technically easier and more beneficial than TTE optimization in terms of time reduction [5]. Furthermore, device-ECG-based auto-AVI adaptation algorithms perform ventricular pacing at a slightly shorter timing than the patient's AVI, appear to be practical, and, in some cases, deliver superior clinical outcomes than echocardiography does [4,6].

The automatic optimization of AVI and VVI for devices has become mainstream, reducing the burden of CRT optimization on physicians. However, evaluating whether cardiac and hemodynamic improvements can be achieved solely through ECG optimization is crucial. Unfortunately, ECG-based optimization alone does not work effectively for some patients, even when using devices with automatic adjustment functions. Notably, optimization through TTE has a significant benefit from the practical viewpoint of directly assessing improvements in cardiac and hemodynamic functions delivered through CRT; however, no single echocardiographic parameter can describe dyssynchrony in its entirety, just as the QRS width [7]. Therefore, evaluating CRT after implantation by combining electrical information from the ECG with mechanical information from the TTE is crucial. However, to date, there are no reports on CRT optimized through combined ECG and TTE. Furthermore, there is currently a lack of evidence demonstrating that echocardiography-guided CRT optimization significantly improves prognosis compared to ECG-based CRT adjustment. In the present study, we compared three time points: the default settings one week after CRT implantation (point A), immediately after CRT optimization using ECG to determine the CRT settings with the narrowest QRS width (point B), and immediately after additional CRT optimization using TTE (point C) to evaluate whether the combination of TTE and ECG optimization would be beneficial.

## Materials and methods

Patients

The study protocol was approved by the Ethics Committee of the Sakakibara Heart Institute. Data from 65 consecutive patients who underwent CRT device implantation for symptomatic congestive HF at Sakakibara Heart Institute, Japan, between March 2018 and July 2022 were retrospectively reviewed. Patients were fully informed about the study and provided consent to participate. The inclusion criteria were as follows, according to the 2013 European Society of Cardiology Guidelines [8]: New York Heart Association (NYHA) functional class II-IV despite optimal medical therapy, LV ejection fraction (LVEF) ≤35%, and a QRS duration ≥130 ms with or without left bundle branch block (LBBB). Patients with persistent atrial fibrillation were excluded due to the highly irregular atrial motion, making accurate assessment of TTE measurements challenging. We assessed the clinical characteristics of patients, including age, sex, body mass index, echocardiographically-derived LVEF, prescription, presence or absence of complete LBBB, chronic kidney disease (estimated glomerular filtration rate <50%), and implantable cardioverter-defibrillator use. The decision to perform either CRT pacemaker or defibrillator implantation was made based on the 2013 European Society of Cardiology guidelines [8].

ECG and TTE data collection

**Figure 1:**
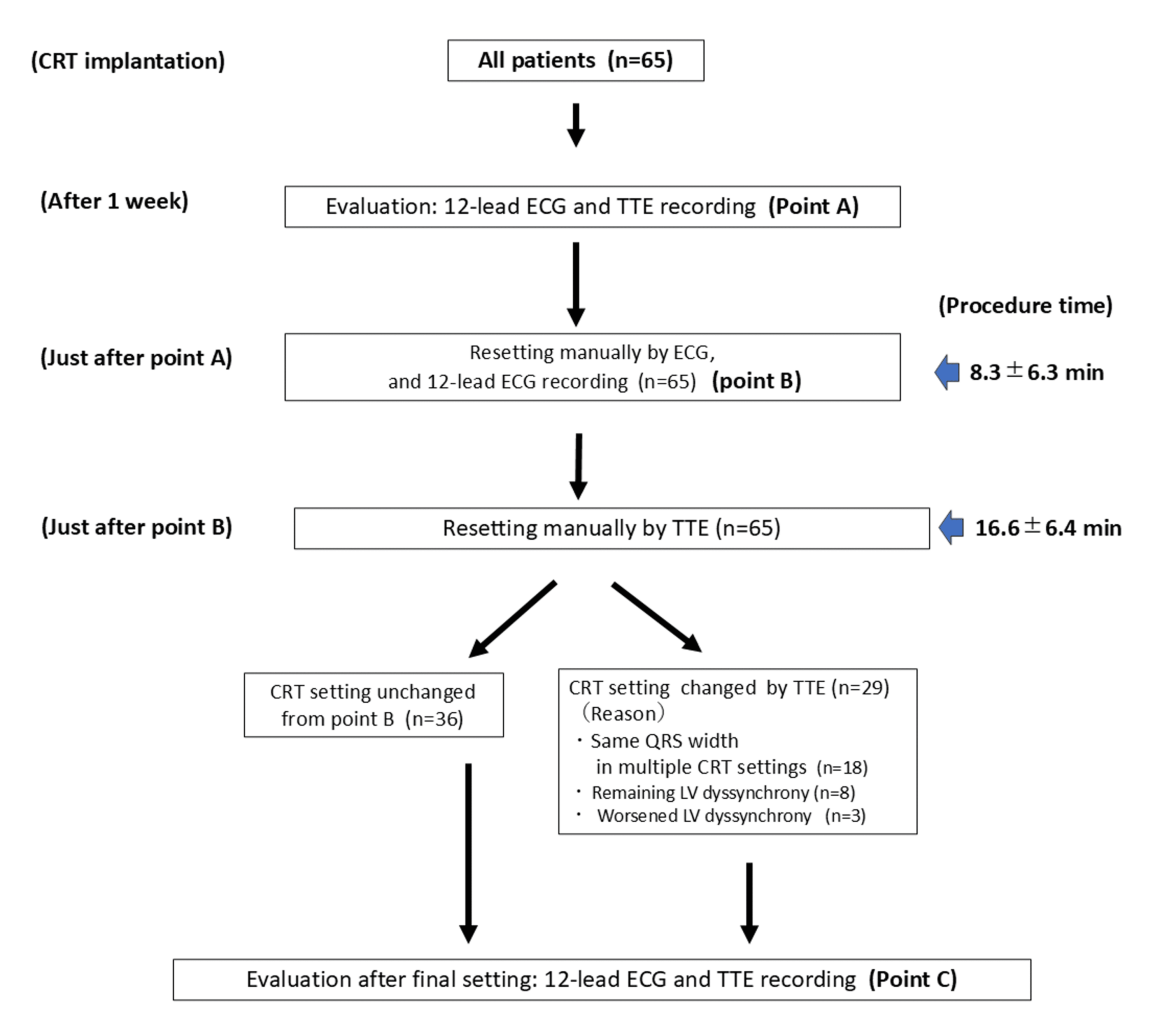
Flowchart of cardiac resynchronization therapy (CRT) optimization method TTE: transthoracic echocardiography; ECG: electrocardiogram; LV: left ventricle

**Figure 2:**
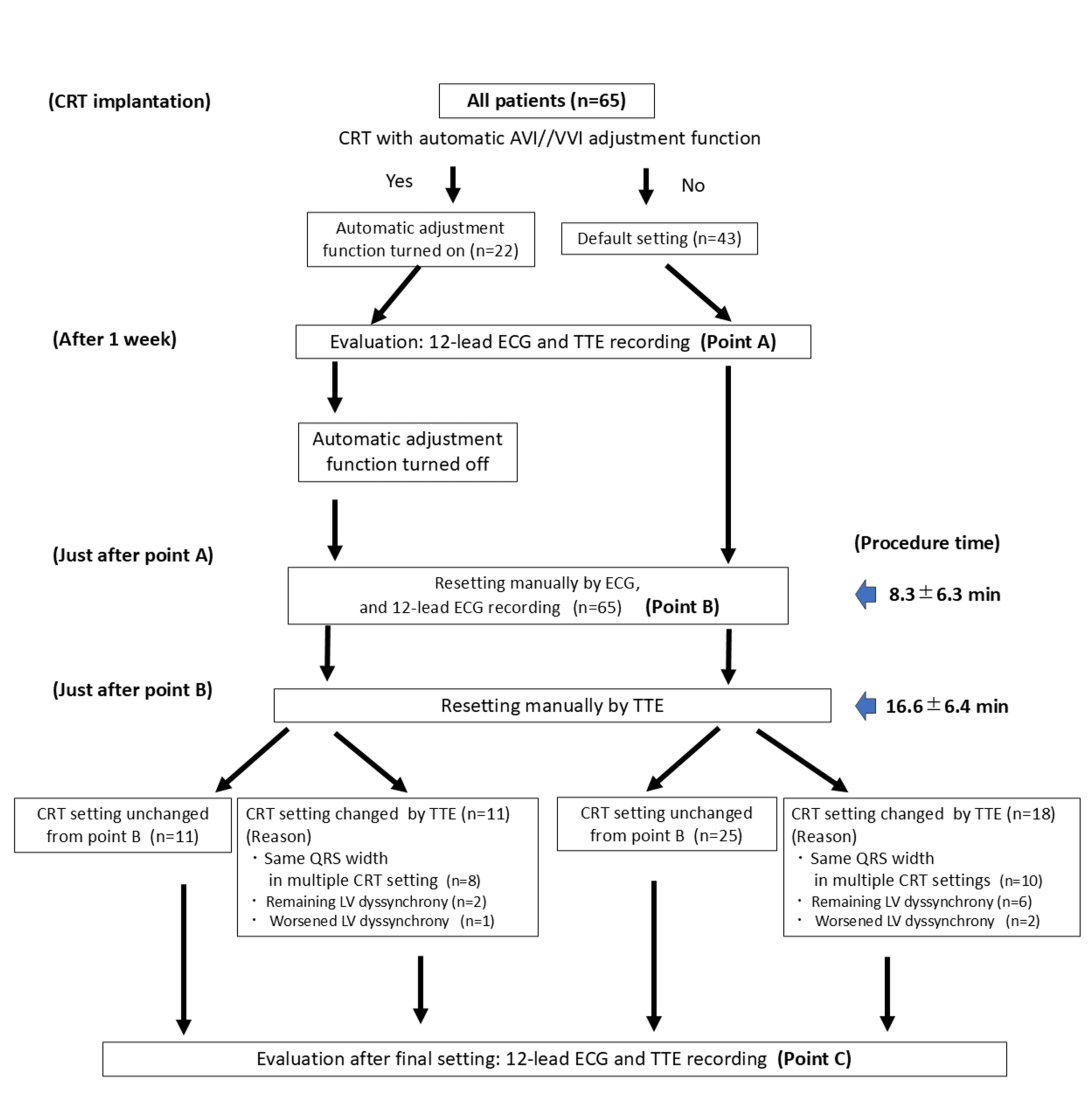
Flowchart of cardiac resynchronization therapy (CRT) optimization for patients with and without automatic adjustment function TTE: transthoracic echocardiography; ECG: electrocardiogram; LV: left ventricle; AVI: atrioventricular interval; VVI: ventriculo-ventricular interval; CRT: cardiac resynchronization therapy

**Figure 3:**
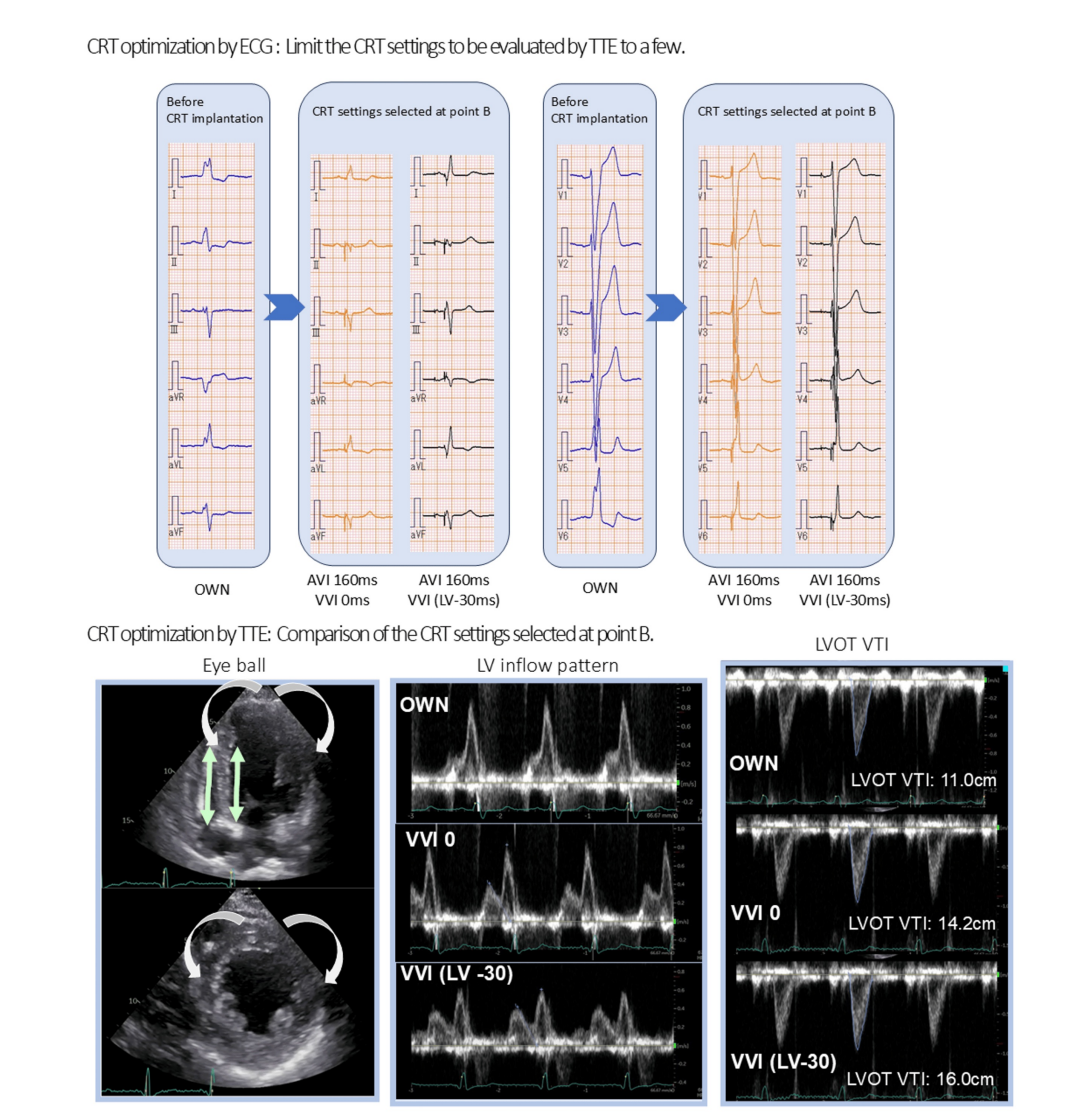
Example of the cardiac resynchronization therapy (CRT) optimization Upper row: The two settings with the narrowest QRS duration selected at point B. The QRS durations of the two settings are equivalent. The electrocardiogram (ECG) at point B shows a shortened QRS duration, compared with pre-CRT implantation. Lower row: CRT optimization with TTE to compare the CRT settings selected at point B. VVI 0: perform LV pacing and RV pacing simultaneously; VVI (LV-30): to precede RV pacing by 30 ms with LV pacing; LVOT VTI: left ventricular outflow tract velocity time integral; TTE: transthoracic echocardiography; LV: left ventricle; VVI: ventriculo-ventricular interval; AVI: atrioventricular interval; OWN: own beat; RV: right ventricular; CRT: cardiac resynchronization therapy

At the time of CRT device implantation, for multipolar LV leads, the pacing point of the LV lead was a good threshold point at a site as far away from the right ventricular (RV) lead as possible on fluoroscopy, with no diaphragmatic stimulation. The CRT AVI/VVI settings were left at default, and this setting was turned on for devices with an automatic AVI/VVI adjustment function. ECG parameters were collected at three time points: one week after CRT device implantation before CRT optimization (point A), after CRT optimization using ECG (point B), and after the final setting using both ECG and TTE when necessary (point C) (Figures 1-2). TTE measurements were performed at points A and C. The QRS duration was measured for all 12 leads, and the narrowest QRS width was selected for the CRT setting (point B). Full-standard echocardiography was performed using a cardiovascular ultrasound system (Vivid E95 Ultrasound System version 203, GE Vingmed Ultrasound, Horten, Norway). The biplane Simpson method was used to measure the LV end-systolic volume, end-diastolic volume, and LVEF. Region-of-interest segmentation was performed for the 18-segment model, and segmentation was automatically performed using analysis software with visual verification and manual correction if needed [8]. Echocardiographic images were analyzed offline using a customized software package (EchoPAC PC Version 203, GE Vingmed Ultrasound, Horten, Norway). The first step in assessing LV function using TTE after CRT device implantation was to evaluate the presence or absence of LV dyssynchrony through an "eyeball" assessment of signs such as septal flash or apical shuffle motion. Furthermore, the severity of the dyssynchrony was quantified using speckle tracking. The LV inflow waveform pattern was also assessed to determine the LV performance in cases with a short AVI if atrial contraction was not interrupted. A Doppler-derived measure of stroke volume from the apical views was obtained using the LV outflow tract velocity-time integral (Figure 3) [9]. LV global longitudinal strain measurements were performed to assess global LV function. Two-dimensional longitudinal strain data were processed using a speckle-tracking examination from apical views, where the reference point was at the beginning of the QRS complex. Through eyeball assessment, at least one experienced physician and an experienced ultrasound technician evaluated intraventricular dyssynchrony parameters, such as apical shuffle or septal flash. In 20 randomly selected echocardiographic studies, the inter- and intra-observer variabilities for LV dyssynchrony parameters were 7% and 5%, respectively. Furthermore, we compared the ECG measurements at points A, B, and C and the TTE parameters between points A and C.

CRT optimization

The flow of CRT adjustment is shown in Figures 1-2. The first optimization of the CRT device was performed using a 12-lead ECG one week after CRT device implantation before discharge. The fusion-optimized interval method was used for CRT adjustment based on ECG, as previously described by Trucco et al. The AV interval was progressively shortened with LV pacing, starting with the longest interval that allowed LV capture and decreasing by 20 ms increments until the interval that produced only LV capture. The 12-lead ECG was recorded for the following three VVI patterns (simultaneous RV and LV pacing (VV 0 ms), LV pre-excitation (30 ms), and RV pre-excitation (30 ms)) at the AVI showing the narrowest QRS, and the CRT setting with the narrowest QRS was selected [10]. If the CRT device had an automatic adjustment function for AVI and VVI, initial measurements (point A) were taken with the automatic adjustment function enabled. The automatic adjustment function was then turned off, and the settings were manually adjusted using the ECG to achieve the narrowest QRS width, as described earlier. The CRT AVI/VVI settings that resulted in the narrowest QRS duration, including those with the automatic AVI/VVI function both on and off, were selected at point B. However, if multiple settings resulted in the narrowest QRS width, all the settings were compared using TTE. For devices without an automatic adjustment function, the parameters immediately before the CRT adjustment were recorded under the default settings for AVI and VVI (point A). After the initial data collection, the CRT was set manually to achieve the narrowest QRS width, and measurements were performed (point B) (Figures 1-2). Immediately after CRT optimization using ECG at point B, the patient’s LV function was re-evaluated using TTE. CRT re-optimization using TTE was performed when the LV inflow pattern was deemed insufficient; for example, the E wave merged too closely with the A wave, the A wave was terminated before the initiation of the QRS complex, and/or a prolonged A wave exceeded the peak of the QRS complex. However, when persistent LV dyssynchrony, such as an apical shuffle or septal flash, was identified through visual inspection, the AVI and VVI of CRT were readjusted using TTE to achieve the best improvement in LV dyssynchrony [9]. However, when the expected improvement in dyssynchrony was achieved at point B, no further adjustments were made using the TTE. After the final CRT optimization using ECG and TTE (point C), we recorded the ECG and TTE parameters (Figures 1-2). We also measured the time required for CRT adjustment using ECG and TTE. Figure 3 shows an example of CRT adjustment. The two settings with the narrowest QRS duration at point B, selected using ECG, were compared using TTE. The evaluation included an eyeball assessment of LV dyssynchrony, LV inflow pattern, and LV outflow tract velocity time integral, with a comprehensive analysis to select the CRT setting that results in a more efficient cardiac output.

Statistical analysis

Categorical variables were presented as the number of patients (%), and continuous variables were expressed as the mean±standard deviation or median. For each variable, differences were evaluated using Pearson’s χ^2^ test for categorical variables and Student’s t-test for continuous variables. The IBM SPSS Statistics for Windows, Version 27 (Released 2020; IBM Corp., Armonk, New York, United States) was used for all analyses, and statistical significance was set at a p-value of less than 0.05.

## Results

Patient characteristics and implantation procedures

All the patients underwent successful device implantation. Table 1 shows the patient characteristics. Of the 65 patients recruited, 2 had their LV lead positioned on the epicardium. However, in the remaining 63 patients, the LV lead was positioned in the lateral vein of the coronary sinus. The mean age of enrolled patients was 74±11 years, and 78.4% were male. NYHA class II was the most common (60%), followed by NYHA class III (33.8%). The major etiology of HF was nonischemic cardiomyopathy (72.3%), and the mean echo-derived LVEF before CRT implantation was low, at 28.1±6.8%. CRT models with an automatic adjustment function were implanted in 22 (29.2%) of the 65 patients (Figure 2), and the remaining 43 patients were implanted with CRT devices without an autonomic adjustment function.

**Table 1:** Baseline characteristics of all 65 patients BMI: body mass index; CLBBB: complete left bundle branch block; CRBBB: complete right bundle branch block; IVCD: intraventricular conduction delay; CKD: chronic kidney disease (estimated glomerular filtration rate <50%); ICD: implantable cardioverter-defibrillator; LVEF: left ventricular ejection fraction; GLS: global longitudinal strain; ACE: angiotensin-converting enzyme inhibitor; ARB: angiotensin II receptor blocker; ARNI: angiotensin receptor neprilysin inhibitor

	Total (n=65)
Age, years	74±11
Male, n (%)	51 (78.4)
BMI, kg/m^2^	21±3.5
NYHA class, n (%)	
Ⅱ	39 (60.0)
Ⅲ	22 (33.8)
Ⅳ	4 (6.2)
CLBBB, n (%)	42 (64.6)
CRBBB, n (%)	3 (4.5)
IVCD, n (%)	20 (30.8)
CKD, n (%)	36 (55.4)
ICD, n (%)	24 (38.5)
Ischemic cardiomyopathy, n (%)	18 (27.7)
Non-ischemic cardiomyopathy, n (%)	47 (72.3)
PR interval before CRT implantation (ms)	165.3±24.1
QRS width before CRT implantation (ms)	192.0±50.3
LVEF, %	28.1±6.8
GLS	6.7±2.9
Apical shuffle, yes (%)	52 (80.0)
Septal flash, yes (%)	48 (73.8)
Beta-blocker, n (%)	60 (92.3)
Antiarrhythmic drug, n (%)	26 (40)
ACE/ARB/ARNI, n (%)	41 (63.1)
Diuretics, n (%)	54 (83.1)

Differences in ECG parameters at points A, B, and C

The average time required for manual optimization of CRT through ECG was 8.3±6.3 minutes (Figures 1-2). Table 2 shows the ECG parameters of all 65 patients at points A, B, and C. On average, the PR interval in the 12-lead ECG was significantly longer at point B than at point A (165±43 ms vs. 146±32 ms, p=0.04), and point C also showed a significantly longer PR interval than point A did (166±38 ms vs. 146±32 ms, p=0.04). However, points B and C did not differ significantly in terms of the PR interval (165±43 ms vs. 166±38 ms, p=0.69). The QRS width was significantly narrower at point A after CRT implantation than before (192.0±50.3 ms vs. 137±20 ms, p<0.01). The QRS width was significantly narrower at point B than at point A (119±20 ms vs. 137±20 ms, p<0.01) and at point C than at point A (124±20 ms vs. 137±20 ms, p<0.01). Conversely, point C showed a slightly wider QRS than point B did (124±20 ms vs. 119±20 ms, p=0.16); however, this difference was not significant (Table 2). The ECG parameters of all 22 patients with the CRT automatic AVI/VVI function showed similar trends to those of all 65 patients; however, no significant differences were observed in the comparison between points A, B, and C (Table 2).

**Table 2:** ECG parameters of all 65 patients at points A, B, and C Point A: before CRT optimization; Point B: after CRT optimization by ECG alone; Point C: final setting using ECG followed by TTE Data are presented as mean±standard deviation. Comparisons between the two points among A, B, and C were made using the Student’s t-test. TTE: transthoracic echocardiography; ECG: electrocardiogram; AVI: atrioventricular interval; VVI: ventriculo-ventricular interval

ECG parameters of all 65 patients at points A, B, and C									
		Point A		Point B		Point C		p-value	
PR interval (ms)		146±32		165±43		166±38		(A) vs. (B) 0.04	
								(A) vs. (C) 0.04	
								(B) vs. (C) 0.69	
QRS width (ms)		137±20		119±20		124±20		(A) vs. (B) <0.01	
								(A) vs. (C) <0.01	
								(B) vs. (C) 0.16	
ECG parameters of all 22 patients whose CRT with automatic AVI/VVI function at points A, B, and C									
		Point A		Point B		Point C		p-value	
PR interval (ms)		154±59		152±21		156±23		(A) vs. (B) 0.91	
								(A) vs. (C) 0.94	
								(B) vs. (C) 0.80	
QRS width (ms)		131±18		125±21		127±22		(A) vs. (B) 0.27	
								(A) vs. (C) 0.51	
								(B) vs. (C) 0.80	

Notably, 36 (55.4%) of the 65 patients did not necessarily need readjustment using TTE because they showed adequate improvement in cardiac function after optimization using ECG (point B), and 29 patients (44.6%) required further adjustment using TTE (Figure 1). Table 3 shows a comparison of the ECG parameters of patients who achieved adequate optimization through ECG-based adjustment alone at point B with those of patients who required further adjustment through TTE. The PR interval in both patient groups tended to be longer at point B than at point A; however, the difference was not significant. Conversely, the QRS width was significantly narrower at point B than at point A in both patient groups (118±21 ms vs. 136±26 ms, p<0.01 in patients who obtained adequate improved dyssynchrony at point B and 120±19 ms vs. 133±18 ms, p=0.01 in the group that did not achieve adequate improvement). However, when comparing the PR interval and QRS width between the two patient groups at points A and B, the two parameters were not significantly different. Furthermore, in patients who achieved unsatisfactory optimization using ECG alone, TEE-based readjustment increased the QRS width to near the level at point A; however, no differences were noted in the final measurements (point C) of ECG parameters between the groups regarding the PR interval and QRS width. There was no significant difference in the baseline clinical data between the patient groups with and without an increase in the QRS width from point B to C (Table 4).

**Table 3:** Comparison of ECG parameters in patients with successful ECG optimization versus those requiring TTE adjustment *The measurements at point C in patients who achieved satisfactory optimization with ECG alone were assumed equal to the results at point B, as these patients did not undergo further adjustment using TTE. Statistical analysis was conducted using the Student's t-test. TTE: transthoracic echocardiography; ECG: electrocardiogram

			Point A	Point B	Point C	p-value (point A vs. B)	p-value (point A vs. C)	p-value (point B vs. C)
PR interval (ms)								
Satisfactory (n=36)			149±39	167±38	167±38	0.11	0.11	-^(*)^
Not satisfactory (n=29)			141±16	159±54	164±41	0.35	0.13	0.81
p-value			ns	ns	ns			
QRS width (ms)								
Satisfactory (n=36)			136±26	118±21	118±21	<0.01	<0.01	-^(*)^
Not satisfactory (n=29)			133±18	120±19	132±16	0.01	0.81	0.02
p-value			ns	ns	ns			

**Table 4:** Comparison of the pre-CRT status between patients whose QRS width increased from point B to point C and those whose QRS width did not increase For each variable, differences were evaluated using Pearson’s χ^2^ test for categorical variables and Student’s t-test for continuous variables. BMI: body mass index; CLBBB: complete left bundle branch block; CRBBB: complete right bundle branch block; IVCD: intraventricular conduction delay; LVEF: left ventricular ejection fraction; CRT: cardiac resynchronization therapy

	QRS width increase from point B to C (n=24)	QRS width did not increase from point B to C (n=41)	p-value
Age, years	75.0±9.2	72.8±11.8	0.43
Male, n (%)	18 (75.0)	33 (80.5)	0.45
BMI, kg/m^2^	21.4±4.2	21.0±3.0	0.65
CLBBB, n (%)	16 (66.7)	26 (63.4)	0.79
CRBBB, n (%)	2 (8.3)	1 (2.4)	0.69
IVCD, n (%)	6 (25.0)	14 (34.1)	0.25
LVEF, %	26.3±7.0	29.1±6.6	0.32
PR interval before CRT implantation (ms)	178.5±28.4	199.2±58.0	0.20
QRS width before CRT implantation (ms)	163.2±25.5	166.6±23.4	0.59

Differences in TTE parameters at time points A and C

In all patients, on average, the LVEF (28.1±6.8% vs. 31.5±8.0%, p=0.01) and septal flash (46.2% vs. 15.4%, p=0.04) significantly improved at point C compared with point A (Table 5).

**Table 5:** TTE parameters in all 65 patients at points A and C A: before CRT optimization; C: final setting using ECG followed by TTE optimization, when necessary Data are presented as mean±standard deviation. For each variable, differences were evaluated using Pearson’s χ^2^ test for categorical variables and Student’s t-test for continuous variables. TTE: transthoracic echocardiography; LVEF: left ventricular ejection fraction; LVEDV: left ventricular end-diastolic volume; LVESV: left ventricular end-systolic volume; LVOT-VTI: left ventricular outflow tract velocity time integral; LV inflow waveform: left ventricular inflow waveform; DcT: deceleration time of E wave at left ventricular inflow; GLS: global longitudinal strain

TTE parameters in 65 patients at points A and C	(A)	(C)	p-value
Heart rate, bpm	69.1±8.8	69.3±8.7	0.92
LVEF, %	28.1±6.8	31.5±8.0	0.01
LVEDV, ml	178.1±69.3	169.3±59.7	0.47
LVESV, ml	129.3±61.2	121.0±54.2	0.44
LVOT-VTI, cm^2^	13.8±4.6	14.7±4.1	0.27
LV inflow waveform			
E-wave	66.7±33.9	65.3±33.1	0.81
A-wave	69.3±22.8	70.9±24.8	0.76
E/A	1.0±1.1	1.0±1.2	0.96
DcT	211.6±73.2	213.9±74.6	0.86
GLS	6.1±3.0	6.5±3.0	0.46
Apical shuffle, yes (%)	42 (64.6)	13 (20)	0.19
Septal flash, yes (%)	30 (46.2)	10 (15.4)	0.04
TTE parameters in all 22 patients with CRT devices with automatic AVI/VVI function at points A and C	(A)	(C)	p-value
Heart rate, bpm	68.0±7.6	66.9±6.2	0.64
LVEF, %	28.0±7.9	32.0±9.2	0.32
LVEDV, ml	188.9±87.8	164.4±59.7	0.24
LVESV, ml	139.4±81.1	113.2±48.4	0.39
LVOT-VTI, cm^2^	13.9±4.2	15.1±3.8	0.39
LV inflow waveform			
E-wave	61.9±32.2	56.0±23.3	0.51
A-wave	67.4±17.4	69.8±20.4	0.71
E/A	0.9±0.5	0.8±0.6	0.85
DcT	214.2±57.9	208.6±45.0	0.74
GLS	6.9±2.5	7.4±3.2	0.65
Apical shuffle, yes (%)	13 (59.0)	6 (27.3)	0.49
Septal flash, yes (%)	8 (36.4)	3 (13.6)	0.04

The average time required for manual CRT optimization using TTE was 16.6±6.4 minutes. Notably, 29 of the 65 patients required CRT resetting with TTE. Among them, 18 had multiple CRT settings with the same QRS duration on ECG, which made it impossible to determine a single CRT setting with ECG alone. Further adjustments based on TTE findings achieved a better setting. LV dyssynchrony remained at point B in eight patients; however, it improved insufficiently, compared with point A. Notably, additional CRT adjustment using TTE improved LV function in all eight patients. Furthermore, three patients exhibited worsened LV dyssynchrony at point B compared with point A and required further CRT adjustment based on TTE (Figure 1). Finally, apical shuffle and septal flash decreased from 21 of 65 patients at point A (75.0%) to 6 (21.4%, p<0.01) at point C and from 17 patients (60.7%) at point A to 5　(17.9%, p<0.01) at point C, respectively (Table 6). There were no significant differences in the baseline characteristics between the groups that required TTE optimization in addition to ECG optimization and those that did not (Table 7).

**Table 6:** TTE parameters at points A and C of the 29 patients who needed CRT optimization with TTE after point B A: before CRT optimization; C: final setting using ECG followed by TTE optimization. For each variable, differences were evaluated using Pearson’s χ² test for categorical variables and Student’s t-test for continuous variables. Data are presented as mean±standard deviation TTE: transthoracic echocardiography; LVEF: left ventricular ejection fraction; LVEDV: left ventricular end-diastolic volume; LVESV: left ventricular end-systolic volume; LVOT-VTI: left ventricular outflow tract velocity time integral; LV inflow waveform: left ventricular inflow waveform; DcT: deceleration time of E wave at left ventricular inflow; GLS: global longitudinal strain

	(A)	(C)	p-value
Heart rate, bpm	69.9±7.2	68.7±6.3	0.54
LVEF, %	28.0±7.6	30.8±8.0	0.18
LVEDV, ml	165.1±68.3	168.9±70.0	0.84
LVESV, ml	121.4±55.6	122.3±61.6	0.96
LVOT-VTI, cm^2^	14.7±5.2	14.6±3.6	0.94
LV inflow waveform			
E-wave	64.6±30.1	63.3±29.1	0.87
A-wave	80.5±30.1	81.7±23.2	0.88
E/A	0.6±0.3	0.6±0.2	0.45
DcT	223.2±83.9	234.2±82.3	0.63
GLS	6.9±3.2	7.4±3.3	0.64
Apical shuffle, yes (%)	21 (75.0)	6 (21.4)	<0.01
Septal flash, yes (%)	17 (60.7)	5 (17.9)	<0.01

**Table 7:** Comparison of baseline characteristics according to the need for TTE optimization in addition to ECG optimization For each variable, differences were evaluated using Pearson’s χ^2^ test for categorical variables and Student’s t-test for continuous variables. TTE: transthoracic echocardiography; BMI: body mass index; CLBBB: complete left bundle branch block; CRBBB: complete right bundle branch block; IVCD: intraventricular conduction delay; LVEF: left ventricular ejection fraction

	Patients with TTE optimization (n=29)	Patients without TTE optimization (n=36)	p-value
Age, years	75.1±9.0	73.2±11.5	0.47
Male, n (%)	21 (72.4)	30	0.57
BMI, kg/m^2^	21.6±4.0	20.8±2.9	0.34
CLBBB, n (%)	21 (72.4)	21 (58.3)	0.49
CRBBB, n (%)	2(6.9)	1 (2.8)	0.92
IVCD, n (%)	6 (20.7)	14 (38.9)	0.17
LVEF, %	26.3±7.3	28.3±6.3	0.27
PR interval before CRT implantation (ms)	186.5±39.6	186.5±39.6	0.53
QRS width before CRT implantation (ms)	167.3±24.4	163.5±24.0	0.54

Comparison of TTE parameters between CRT devices with and without automatic adjustment function

Twenty-two patients (34.8%) received CRT implants with an auto-adjustment function for optimization and 43 (65.2%) received CRT without this function (Figure 2).

Among the 43 patients who received CRT without autonomic adjustment function, 25 patients (58.2%) achieved appropriate improvement in LV function, and 18 (41.8%) needed additional adjustment with TTE after ECG adjustment. Notably, 10 of the 18 patients (55.5%) required TTE because the multiple settings of the QRS width were observed with ECG adjustment alone, six needed additional TTE adjustment due to insufficient improvement of LV dyssynchrony, and two showed worsened LV dyssynchrony after ECG setting.

Notably, 11 (50%) of the 22 patients who received CRT devices with autonomic adjustment functions required additional CRT optimization using TTE after ECG optimization. No significant difference was observed in the frequency necessary for additional CRT optimization through TTE between the CRT group with (50%) and without (42%) automatic AVI/VVI adjustment function (p=0.61). However, among the 11 patients who required readjustment through TTE, eight (72.7%) required TTE because multiple settings with the same QRS width were observed during ECG adjustment alone. Two patients had to undergo TTE optimization because significant LV dyssynchrony remained at point B; however, partial improvement of the dyssynchrony was observed, compared with point A. Readjustment using TTE further improved LV dyssynchrony. Another patient required TTE-based CRT readjustment because LV dyssynchrony worsened at point B compared with point A (Figure 2). Among the 22 patients who received CRT with automatic AVI/VVI adjustment function, a comparison of TTE parameters at points A and C showed similar trends to those observed in the overall group of 65 patients. Notably, septal flash significantly improved at point C compared with point A (36.4% vs. 13.6%, p=0.04) (Table 5).

## Discussion

Major findings

In this study, we evaluated the effect of CRT optimization using both ECG and TTE adjustments. Initially, CRT was optimized with ECG, allowing the selection of additional optimal settings in a limited number of cases.

Using TTE, we further optimized the dynamic atrioventricular coordination and intraventricular synchronization, which could not be achieved using ECG optimization alone (Figures 1-2). Our results showed that approximately half of the patients (29 of the 65 patients, 45%) who underwent CRT implantation needed additional optimization using TTE to achieve an adequate setting after adjustment using ECG. Notably, the frequency of additional optimization using TTE did not differ between patients with and without automatic adjustment function devices; 50% of the patients were implanted with an automatic adjustment function, and 42% of those without this function required further adjustment using TTE. These results suggest that, even with an automatic adjustment function, additional TTE-guided CRT optimization can improve LV dyssynchrony in some patients.

CRT optimization using ECG

Our results showed that, compared with point A (146±32 ms), the PR intervals at both points B (165±43 ms, p=0.04) and C (166±38 ms, p=0.04) were significantly prolonged in all 65 patients. The conventional default settings for AVI were 100-200 ms and 130-150 ms for sensed and paced AV, respectively, depending on the manufacturer. However, these settings may not always be optimal for some patients. Therefore, in patients who do not achieve satisfactory improvement with ECG optimization alone, AVI optimization based on echocardiographic determination of the optimal diastolic filling is useful. The best way to take advantage of atrial function is to have an atrial contraction end just before the start of an LV contraction. On TTE, the atrial wave of the LV inflow velocity waveform ends immediately before the mitral valve closure. Patients undergoing CRT implantation often have intra-atrial conduction delays after atrial pacing [11], resulting in a delay between A pace and mechanical atrial contraction, which reduces the effectiveness of the default AVI setting in utilizing atrial function. Under conventional default settings, the PR interval may be too short for some patients, resulting in an atrial wave of the LV inflow velocity waveform ending after mitral valve closure. In our study, the PR interval in 29 patients (44.6%) who required additional CRT optimization using TTE tended to be longer at point C than at point B (Tables 2-3). These findings support the report by Kedia et al., in which AVI was prolonged compared with that before AVI optimization was performed using TTE [12].

Another critical factor in determining the effectiveness of CRT is the QRS width, with narrower widths resulting in greater clinical benefits [13,14]. A narrow QRS suggests well-synchronized ventricular activation and contraction, resulting in improved cardiac performance. Our data showed that the QRS width was significantly narrower at points B (119±20 ms, p<0.01) and C (124±20 ms, p<0.01) than at point A (137±20 ms) in 65 patients (Table 2). This was also observed in the patients who achieved satisfactory improvement at point B (Table 3). However, in 29 patients who required CRT readjustment after adjustment using ECG at point B, the QRS width after the final adjustment using TTE (point C) increased and returned to the level at point A.

Furthermore, no significant difference was observed in the QRS width between point A and after the final optimization (point C) (Table 3). This suggests that in patients who did not achieve optimal CRT adjustment using ECG alone, the QRS width could not be a sufficient indicator of better clinical results; however, overall, a narrower QRS width tends to improve cardiac function. Therefore, today, CRT with auto-adjustment of AVI and VVI functions based on the QRS width is the mainstream approach for new devices. However, CRT with this function fails to reach the expected clinical results in some patients with severe HF. Therefore, it should be emphasized that TTE may be necessary to improve LV dyssynchrony regardless of the QRS width. This may explain why the automatic adjustment function does not exclude the efficacy of TTE readjustment for appropriate CRT optimization. As shown in Table 7, no factors that could predict the need for further CRT adjustment with TTE were identified in the pre-CRT implantation data. Therefore, it may be important to actively assess LV dyssynchrony using TTE in the early postoperative period.

The PR interval and QRS width did not differ between patients with and without satisfactory optimization at points A and B, indicating that these two ECG parameters could not separate patients with better results from those with insufficient improvement (Tables 3, 7).

CRT optimization using TTE

Using the LV inflow waveform pattern of TTE for CRT optimization has been proven safe, feasible, and effective in reducing filling pressures and improving cardiac output, leading to a better hemodynamic profile and reducing adverse events [9]. Mullens et al. reported several cases with narrowed QRS on ECG after CRT device implantation; however, mechanical inappropriateness remained [15]. Notably, various CRT configurations (13-30 patterns) have been created with the introduction of quadrupole LV leads and multipoint pacing. However, assessing these configurations using TTE is time-consuming and challenging, particularly in patients with low cardiac function, as it can lead to worsening HF or atrial fibrillation during the process. To minimize the burden on patients, CRT optimization should be performed as quickly as possible.

To address this, we first used ECG to optimize CRT and reduce the number of configurations that required evaluation using TTE. This approach reduced the time for CRT optimization using ECG and TTE to within 30 min (8.3±6.3 minutes for ECG and 16.6±6.4 minutes for TTE optimization), making it practical for clinical use. As a result, LV function showed significant improvement with appropriate CRT optimization using both ECG and TTE, with LVEF increasing from 28.1±6.8% to 31.5±8.0% (p=0.01) in patients who received TTE optimization (Table 5). Twenty-nine patients who required CRT optimization with TTE after point B showed more noticeable improvement in LV dyssynchrony, such as apical shuffles and septal flashes (Table 6).

Furthermore, some patients required re-optimization using TTE after point B, regardless of the presence of CRT with an auto-AVI/VVI function (Figure 2), and the frequency of need for re-optimization did not differ between CRT with and without an automatic optimization function. This again suggests the limitation of CRT optimization using ECG alone in obtaining the expected results.

Limitations

Our study has some limitations. First, this was a retrospective single-center study with a small sample size. Therefore, a large-scale prospective study is required. Second, in this study, we evaluated only short-term results. The long-term clinical effects require further study. The impact of LV lead electrode position on therapeutic response was not assessed in this study. CRT response may be influenced by various factors, including the position of the lead electrode. Third, the evaluation was conducted only at rest. Currently, CRT with AVI and VVI auto-adjustment functions has been introduced. This helps improve diastolic filling time, which an increased heart rate may compromise through atrial pacing and provides real-time AVI adjustment in response to heart rate fluctuations during exercise [16]. Chen et al. reported that CRT with an AVI auto-adjustment function demonstrated better electromechanical resynchronization than CRT with a fixed AVI did [17]. The automatic adjustment function reduces the CRT optimization time for physicians [18]. Notably, some reports suggest that this function leads to a better long-term prognosis compared with that in TTE-adjusted groups [19]. However, in this study, even with CRT devices equipped with the automatic AVI/VVI function, further CRT adjustment led to better outcomes in cardiac function, suggesting that additional investigation with a larger number of patients is required. Fourth, TTE-based CRT optimization involves subjective parameters, such as apical shuffle and septal flash, and requires the integration of multiple indices, which necessitates clinical experience for proper evaluation, thus posing a potential limitation. Therefore, if no significant difference is observed in LV dyssynchrony on TTE between manual and automatic adjustments using the device, automatic adjustment may be a safe choice for patients during exercise [6]. Recently, CRT devices that optimize AV delay based on heart sound measurements have emerged. These devices utilize hemodynamic indicators beyond QRS width, and their future potential is worth looking forward to [19]. In the present study, we found that ECG adjustment alone can improve LV dyssynchrony in many cases; however, there are instances where it may worsen the condition after CRT optimization using ECG alone, even in the acute phase. In such cases, further evaluation using TTE is necessary. Additionally, manual adjustment requires more effort, and the use of ECG and TTE for CRT optimization can lead to better improvements in LV dyssynchrony.

## Conclusions

Notably, some patients who received CRT could not gain an expected improvement in LV dyssynchrony through ECG-based optimization alone, even for CRT with an automatic adjustment function. However, additional adjustments using TTE could overcome, at least in part, this problem. CRT adjustment using ECG, followed by TTE when necessary, is an efficient way to improve LV dyssynchrony in a time-saving manner. The combination of these two techniques has proven to be a highly successful approach to enhancing heart function.
